# sRNA Antitoxins: More than One Way to Repress a Toxin

**DOI:** 10.3390/toxins6082310

**Published:** 2014-08-04

**Authors:** Jia Wen, Elizabeth M. Fozo

**Affiliations:** Department of Microbiology, University of Tennessee, M409 Walters Life Sciences, Knoxville, TN 37996, USA; E-Mail: jwen@vols.utk.edu

**Keywords:** type I toxin-antitoxin, type III toxin-antitoxin, small RNA, small peptide

## Abstract

Bacterial toxin-antitoxin loci consist of two genes: one encodes a potentially toxic protein, and the second, an antitoxin to repress its function or expression. The antitoxin can either be an RNA or a protein. For type I and type III loci, the antitoxins are RNAs; however, they have very different modes of action. Type I antitoxins repress toxin protein expression through interacting with the toxin mRNA, thereby targeting the mRNA for degradation or preventing its translation or both; type III antitoxins directly bind to the toxin protein, sequestering it. Along with these two very different modes of action for the antitoxin, there are differences in the functions of the toxin proteins and the mobility of these loci between species. Within this review, we discuss the major differences as to how the RNAs repress toxin activity, the potential consequences for utilizing different regulatory strategies, as well as the confirmed and potential biological roles for these loci across bacterial species.

## 1. Introduction

For many years, proteins were considered the master regulators of gene expression, and RNA was seen only as the “intermediate” between the genetic code DNA, and proteins, the functional moieties of the cell. However, increasing numbers of RNAs serving in regulatory capacities were identified over the years. Within the past two decades, we have entered into a new RNA regulatory era where countless examples of RNAs that function to control gene expression across all kingdoms of life have been characterized.

**Table 1:** Features of described type I and type III toxin-antitoxin loci.

Locus		Founding member (plasmid or bacterium) ^a^	Genetic organization ^b^	Mode of antitoxin action ^c^
Type I	Hok/Sok	Plasmid R1 (63)	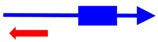	Inhibit protein synthesis
	RNAI/RNAII	pAD1 (48)	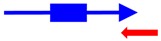	Inhibit protein synthesis
	Ldr/Rdl	*Escherichia coli* (34)	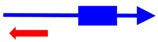	Inhibit protein synthesis
	TisB/IstR1	*Escherichia coli* (39)	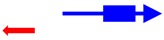	Inhibit protein synthesis
	TxpA/RatA	*Bacillus subtilis* (15)	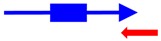	Stimulate mRNA degradation
	SymE/SymR	*Escherichia coli* (55)	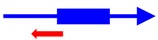	Inhibit protein synthesis
	Ibs/Sib	*Escherichia coli* (41)	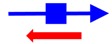	Inhibit protein synthesis
	ShoB/OhsC	*Escherichia coli* (41)	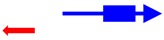	Inhibit protein synthesis
	BsrG/SR4	*Bacillus subtilis* (58)	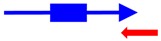	Stimulate degradation and inhibit translation
	Zor/Orz	*Escherichia coli* (5, 43)	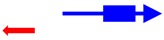	Inhibit protein synthesis
	RalR/RalA	*Escherichia coli* (56)	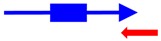	Inhibit protein synthesis
	DinQ/AgrB	*Escherichia coli* (42)	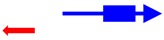	Inhibit protein synthesis
Type III	ToxN/ToxI	pECA1039 (59)	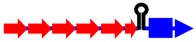	Protein sequestration
	ABIQ/antiQ	pSRQ900 (62)	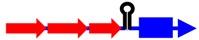	Protein sequestration

^a^ The founding member (first description) of each toxin-antitoxin locus is indicated. Note that many homologs to these systems have been identified and characterized; the text gives details of those. ^b^ The blue arrow represents the toxin mRNA; the blue box, the toxin coding region. The red arrow indicates the antitoxin; note for the type III antitoxins, they consist of repetitive sequences. ^c^ The mode of action of the antitoxin has not been validated for all; the reader is encouraged to examine the text for those details.

Some of the earliest described examples of RNA regulation came from studies examining the control of plasmid replication. Later studies identified toxin-antitoxin loci on plasmids. The toxin-antitoxin loci consist of two genes: one encodes a protein whose overexpression is toxic to bacterial cells and the other encodes an antitoxin that functions to repress toxin gene expression or its activity [1]. These gene pairs are often co-transcribed or encoded antisense to each other on the opposite DNA strand. The earliest studies of these loci determined that they were responsible for “plasmid addiction” [2,3,4]. Essentially, the toxin gene products were highly stable and the antitoxin gene products were highly unstable. Upon cell division, if a daughter cell did not inherit the plasmid, the unstable antitoxin would be degraded, while the toxin was not. The toxin was then able to kill the plasmid-less daughter cell. However, if the cell inherited the plasmid, the unstable antitoxin could be replenished, and prevent killing of the cell by the toxin. Recently, homologs to the different described plasmid systems (see below) have been found on the chromosomes of bacteria, and some newly identified loci have been found on chromosomes with no apparent homology to mobile genetic elements [5,6,7,8,9,10,11]. Thus, the biological function of the chromosomal loci may be different than those found on plasmids, and new data (discussed below) supports this idea.

There are several different described classes of toxin-antitoxin systems. These classification schemes are based on the type of antitoxin (either RNA or protein) and how the antitoxin functions to control toxin expression or activity. A recent review [1] summarizes these classes, and gives a general overview of the function of these loci. For the purpose of this review, we will focus solely on the type I and type III loci in which the antitoxin is a regulatory RNA. For the type I loci, the antitoxin RNA has sequence complementarity to the toxin mRNA, and through base pairing interactions, it either inhibits translation and/or stimulates degradation of the mRNA. For the type III loci, the RNA antitoxin binds to the toxic protein, sequestering it or blocking its biochemical activity. Table 1 provides a summary of the general features of the major type I and type III loci described to date.

Within this review, we will discuss the nuances as to how the RNA antitoxins regulate toxin expression and/or function, as well as the potential benefits for using one repressive method over the other. Additionally, we will report on some recent progress made in the attempts to understanding the biological function of these loci.

## 2. Repression by RNA Antitoxins and Toxin Function

### 2.1. Type I Antitoxins: Repression through Base Pairing Interactions

As stated, type I antitoxins have sequence complementarity to their cognate toxin mRNAs. This complementarity can be as little as 18 nt of perfect matching to more than 75 nt of perfect matching, though not all of the sequence complementarity may be needed for repression. The majority of type I antitoxins are encoded *cis* to their targets, directly antisense to the toxin gene and not in another chromosomal (or plasmid) location. However, other regulatory RNAs in bacteria (referred to as small RNAs or sRNAs) that act via base pairing are not encoded directly antisense to their targets and so they often have limited complementarity to their targets (6–12 nts). Many of these regulatory RNAs found in *Escherichia coli* require the protein Hfq to stabilize their interactions with their target mRNAs, though the requirement for Hfq can vary in other species [12,13]. The well characterized type I antitoxins do not require Hfq for function, and this is likely due to the increased base pairing interactions between the antitoxins and the toxin mRNAs.

As a result of the formation of the RNA duplex between the toxin mRNA and antitoxin RNA, two main outcomes typically occur: toxin mRNA degradation can be stimulated or its translation can be inhibited. Within this section, we will highlight some key examples of systems that use these mechanisms.

#### 2.1.1. Repressing Type I Toxins: Controlling mRNA Stability

**Figure 1:**
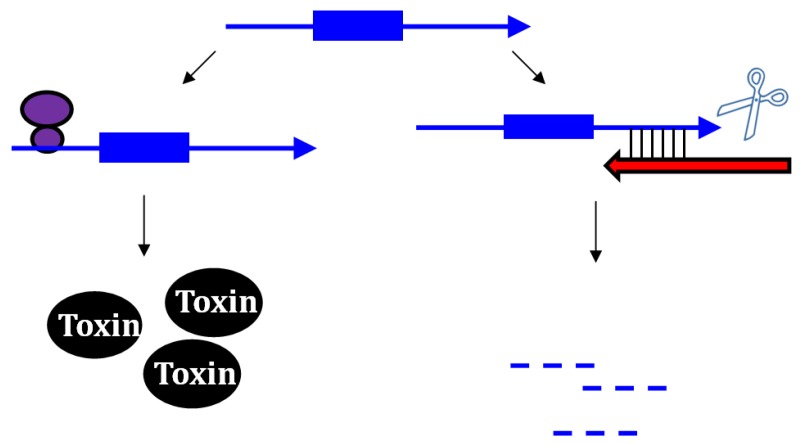
Repression of *txpA* expression by stimulation of mRNA degradation. The *txpA* toxin mRNA (blue) can be translated by the ribosome (purple) to produce the toxic protein. However, if the antitoxin RatA (red) is present and interacts with the toxin, RNase III can cleave the double-stranded complex, thereby initiating mRNA degradation which eventually prevents toxin translation.

Upon the formation of an RNA duplex, the bacterial endoribonuclease, RNase III, encoded by the *rnc* gene, will often cleave the double-stranded RNA (as illustrated by *txpA/ratA* in Figure 1). While most type I antitoxins examined to date may stimulate RNA degradation, their primary mode of action appears to be through inhibition of mRNA translation. However, the *txpA*/*ratA* locus of *Bacillus subtilis* appears to function primarily through mRNA degradation [14,15].

The *txpA*/*ratA* locus was first identified in *B. subtilis* through microarray analysis during a search for novel genes within intergenic regions [15,16]. The locus is located within the *skin* element, a genetic element that is excised from the chromosome upon sporulation. Northern analysis confirmed the presence of two converging transcripts that overlapped each other by approximately 75 nt within this locus [15]. The one gene, *txpA*, encoded a 59 amino acid peptide; the other, *ratA*, did not contain a potential open reading frame. Given this and other hallmarks of the locus, the authors proposed it functioned similar to a type I toxin-antitoxin locus. Indeed, overproduction of the toxin-encoding gene, *txpA*, from an inducible promoter was highly toxic to *B. subtilis*, but co-expression of the RatA RNA could prevent the toxicity [15]. Additionally, a strain deleted for *ratA* exhibited a lysis phenotype on agar plates after several days of growth [15], indicating the importance of the antitoxin to prevent aberrant expression.

Repression of *txpA* by RatA was likely not due to translation inhibition as there was no sequence overlap or complementarity between the antitoxin and the toxin coding region or translation initiation region. Thus, it was hypothesized that the interactions with their 3' ends could stimulate degradation of the toxin mRNA. This was supported by deletion of either *ratA* [15] or *rnc* (encodes RNase III) [14]: deletions of either gene led to elevated levels of the *txpA* mRNA in comparison to the wild type strain. Further support of this model came from *in vitro* interaction studies. Upon interaction with RatA, the *txpA* mRNA undergoes several structural changes that lead to the formation of new RNase III sites. Thus, the current data supports that the major effect of RatA is to destabilize *txpA* mRNA, and not to impact its translation [14].

#### 2.1.2. Repressing Type I Toxins: Controlling Protein Synthesis

The majority of type I antitoxins function through inhibition of toxin mRNA translation. However, not all antitoxins simply base pair over the toxin ribosome binding site or start codon. Toxin mRNA translation is often tightly regulated, even without the presence of the antitoxin. For many, the toxin’s ribosome binding site is sequestered within a tight secondary structure, and translation requires either processing events or other elements in order for it to occur. In fact, many type I antitoxins directly interfere with those other translational elements to control toxin expression.

**Figure 2:**
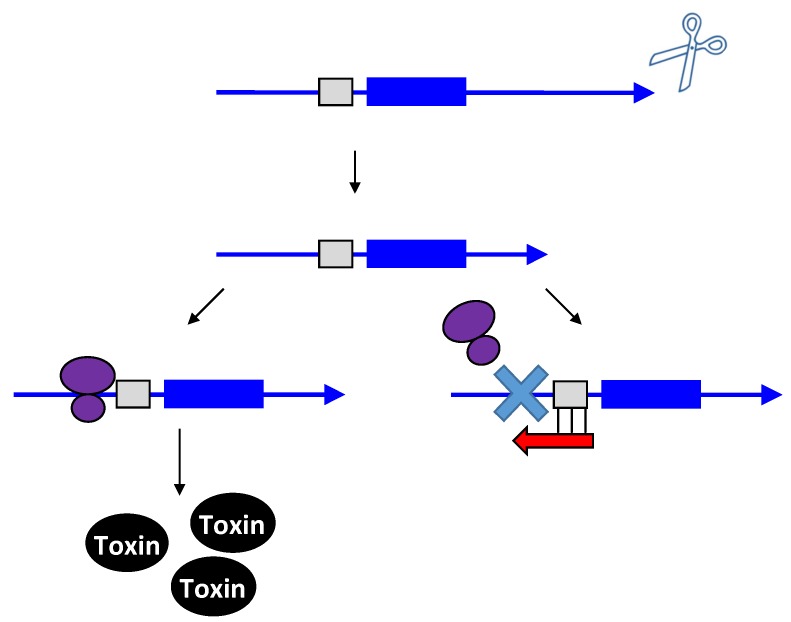
Repression of *hok* translation by Sok. The *hok* mRNA (blue) must first be processed at its 3' end prior to interaction with either the ribosome (purple) or Sok (red). Translation of the *hok* mRNA is dependent upon translation of an upstream open reading frame *mok* (grey) by the ribosome. Binding of the Sok antitoxin RNA (red) blocks translation of the leader peptide, thus preventing toxin expression.

The first type I toxin-antitoxin locus to be identified and thoroughly characterized is the *hok*/*sok* locus of plasmid R1 found within *E. coli* and related Gram-negative species. Initially, a region within the plasmid deemed the *par* locus was determined to be responsible for plasmid maintenance; later it was shown that the locus encoded a toxic protein (Hok) and an RNA antitoxin, Sok. The *hok* mRNA has an exceptionally long 5' untranslated region (UTR), and the region of complementarity to Sok is within the long 5' UTR. The long 5' UTR folds into a highly structured RNA that neither the antitoxin nor ribosome can access [17,18,19]. Upon cleavage of the *hok* mRNA at its 3' end, the mRNA refolds and Sok can access the region of complementarity [18,19,20]. Yet, how could Sok regulate expression if the region of base pairing is far from the *hok* translation initiation codon and the ribosome binding site? Within the *hok* long 5' UTR is another open reading frame termed *mok*. Translation of *mok* is absolutely required for translation of *hok*; the ribosome translates *mok*, and then can translate *hok* [21]. The region of complementarity to Sok overlaps *mok*, thus binding of Sok blocks translation of both *mok* and *hok* (Figure 2). The RNA duplex is then subject to degradation by RNase III. However, *hok* could still function in plasmid maintenance in an *rnc*-strain, providing additional support that a block in translation is the major mechanism of Sok repression [22]. RNase III activity though is critical to prevent future translation of *hok* mRNA; upon cleavage by the ribonuclease, the *hok* mRNA is untranslatable.

The *hok*/*sok* locus was shown to have homologs in a variety of plasmids. These homologs include the *flm* and *srnB-srnC* loci of the F plasmid, and the *pndA-pndB* locus of the plasmid R483. Gene arrangement, cell killing, and some of the regulatory features examined in these systems are consistent with the *hok*/*sok* findings [23,24,25,26,27,28,29,30]. Along with the homologs in other plasmids, copies of the *hok*/*sok* locus have been discovered on bacterial chromosomes [5,6,31], but debate lingers regarding their functions.

Control of the Ldr toxins by the Rdl antitoxins is thought to act in a similar fashion to *hok*/*sok* [32]. The LDR repeat is a large sequence found four times within the *E. coli* MG1655 chromosome [33,34,35]. Three of the repeats are in tandem to each other, and the fourth is found directly opposite on the chromosome. Within each repeat are two genes: one encodes Ldr, a small 35 amino acid protein, and the second, a small antisense RNA, Rdl, of about 60 nt [34]. Overproduction of LdrD was toxic to *E. coli*, yet co-expression of its cognate antisense RNA RdlD, inhibited the toxicity.

The mechanism used by RdlD to regulate *ldrD* was not immediately clear. Like *hok*, the *ldrD* mRNA possesses a long 5' UTR, and the antitoxin is encoded so that it overlaps the 5' UTR and not the ribosome binding site or the start codon [34]. Gerdes and Wagner proposed that RdlD regulates in a manner similar to Sok; they identified a small open reading frame, *ldrX*, within the 5' UTR of *ldrD* [36]. They proposed that translation of *ldrD* is dependent upon translation of *ldrX*, and that base pairing by RdlD would block translation of both *ldrX* and *ldrD*. Further work, though, is needed to confirm this hypothesis.

Type I antitoxins can also block toxin mRNA translation through other mechanisms. One example is repression of *tisB* expression by IstR-1. These genes are encoded divergent from each other in the *E. coli* chromosome and were identified in searches for small RNA genes and for LexA binding sites within the *E. coli* chromosome [37,38,39]. LexA is a transcriptional repressor of genes involved in the DNA damage response (known as the SOS response). DNA damage induced transcription of *tisB*, but not *istR-1* [39]. Deletion of either *tisB* or *istR-1* had no obvious effects on *E. coli*; however, the *istR-1* deletion could not be moved into a LexA deficient strain. In this strain, *tisB* expression is higher than in a wild type strain as there is no longer transcriptional repression. The group identified that *tisB* encoded a 29 amino acid long hydrophobic protein whose overproduction was highly toxic [39]. Examination of the *istR-1* sequence revealed that it possessed 21 nt of complementarity to the 5' UTR of the *tisB* mRNA, and overexpression of IstR-1 could block TisB-induced toxicity, explaining the inability to delete *istR-1* in a LexA deficient strain. Similar to other described toxin-antitoxin pairs, *tisB* mRNA levels were elevated in strains deleted either for *rnc* or *istR-1* [39]. This strongly suggested that formation of the *tisB-*IstR-1 RNA duplex triggered degradation of *tisB* by RNase III.

**Figure 3:**
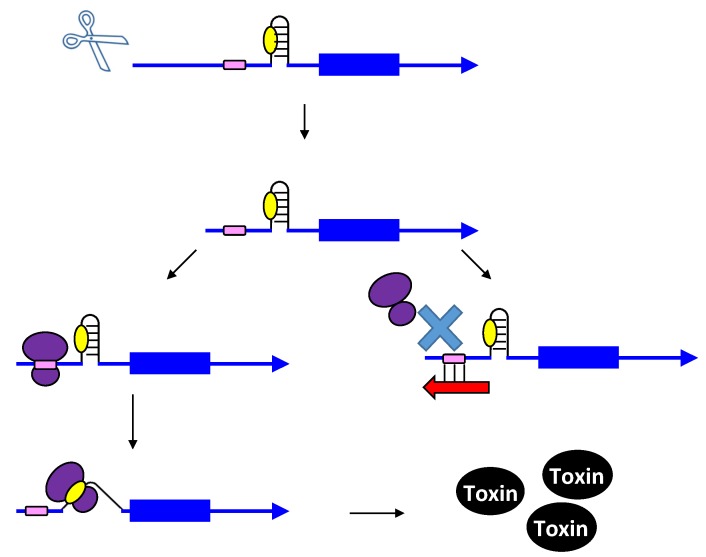
Repression of *tisB* translation by IstR-1. Translation of *tisB* mRNA is dependent on both a processing event and binding of the ribosome (purple) to a standby site (pink) as the real toxin ribosome binding site (yellow) is sequestered in a secondary structure. First, the full-length *tisB* mRNA (blue) must be processed at its 5' end in order for IstR-1 (red) or the ribosome to bind. Following processing, the standby site (pink) is accessible to the ribosome and the antitoxin (red; IstR-1). Binding by the antitoxin will block binding of the ribosome to the standby site. In the absence of the antitoxin, the ribosome can bind to the standby site and then onto the true ribosome binding site (yellow).

Given that IstR-1 bound far from the ribosome binding site and start of *tisB* translation, could it block *tisB* expression? *In vitro* studies elucidated a unique regulatory system, distinct from what was described for the *hok*/*sok* locus [40]. The full-length *tisB* mRNA must be cleaved at its 5' end in order for it to be translated or interact with IstR-1. This shorter form of *tisB* has an altered structure from the full-length mRNA: the shorter form possesses a stretch of single-stranded nucleotides where complementarity to IstR-1 is found. Toeprints identified that ribosomes bind to this unstructured region known as a standby site. After binding to the standby site, the ribosome can move to the true *tisB* ribosome binding site and begin translation of the toxin (Figure 3). Yet, the ribosome is in competition with IstR-1 to bind at the standby site [40]. Thus, translation of *tisB* requires cleavage of the full-length mRNA, structural rearrangements, and the positioning of the ribosome, and not IstR-1, at the standby site.

While repression of *tisB* by IstR-1 is distinct from the repression of *hok* by Sok, the *tisB* model may be common for other “non-traditional” Type I chromosomal systems where the toxin and antitoxin genes are not encoded directly opposite each other on the chromosome. The *tisB* and *istR-1* genes are encoded divergent from each other; three additional genetic loci identified within *E. coli* match this gene arrangement: *shoB-ohsC*, *zor-orz* and *dinQ-agrB* [5,41,42,43]. These loci are similar in that the divergently transcribed antitoxins are complementarity to a region within the long 5' UTR of their corresponding toxin mRNAs. These antitoxins do not overlap the translation initiation site or coding sequence. It is possible that these antitoxins repress their toxins by binding to a standby site like IstR-1 or by binding over another translational element, like Sok. Like *hok*/Sok and *tisB*-IstR-I, the *zorO*-OrzO complex is cleaved by RNase III and RNase III is also required for suppression of ZorO-induced toxicity [43]; further analysis of the other systems is needed to confirm if they are also subjected to RNase III processing.

Translational repression is also exemplified in the *par* locus of the pAD1 plasmid of *Enterococcus faecalis*. This was the first type I locus to be identified and described in a Gram-positive bacterium. Initially, a locus deemed *par* was found to be required for stability of the pAD1 plasmid [44,45]. Two converging RNA transcripts referred to as RNA I and RNA II were identified in the locus; work later demonstrated that RNA I encodes a small, hydrophobic toxic protein, Fst [44,46,47]. Expression of RNA II was needed to repress Fst toxicity. The converging RNAs overlap at their 3' ends and share a terminator; however, this overlap would not extend into the coding region [44,45,48].

Although RNA I and RNA II are arranged genetically like *txpA-ratA* with extensive overlap in their 3' ends, repression of RNA I by RNA II occurs through translational inhibition. Within RNA I and RNA II are direct repeats known as DRa and DRb. Through careful *in vitro* analysis, it was shown that these repeats interact via base pairing and successfully block binding of the ribosome to RNA I, thus preventing its translation [48,49]. *In vitro*, this RNA duplex is incredibly stable [50]; however, *in vivo* analysis shows that the half life of the RNA II antitoxin is shorter than that of RNA I [48]. Thus, additional unknown *in vivo* factors are needed to disrupt the RNA duplex to cause degradation of RNA II. Homologs of the RNA I/RNA II locus have been found within many bacterial chromosomes [7,51,52,53], and analysis of some of those systems implies that they are regulated in the same fashion as was originally described for the pAD1 locus.

**Figure 4:**
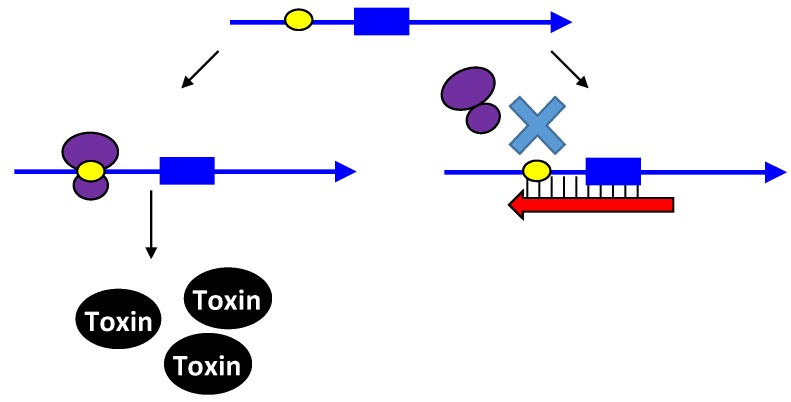
Repression of *ibs* by the Sib antitoxin. The *ibs* mRNA (blue) is translated by the ribosome (purple) when the Sib antitoxin (red) is not present. Binding of the antitoxin RNA (red) blocks translation as it overlaps the ribosome binding site (yellow) and the translation initiation codon and then can potentially extend over the entire coding region.

Other type I antitoxins also effectively block ribosome binding to the toxin mRNA or translation initiation of the toxin mRNA as their major means to prevent protein expression. One example is the Ibs-Sib locus initially described in *E. coli* MG1655 [41]. This locus is found in five copies in the genome of MG1655 and was initially noted as a 140 nt long repeat sequence [35]. The Sib antitoxins overlap the entire coding region and the ribosome binding site of the toxic *ibs* mRNA (Figure 4). An *in vitro* study showed that the formation of such a complete and extensive duplex was not needed for repression. Two recognition domains, TRD1 and TRD2, were identified as critical for RNA-RNA interactions [54]. TRD1 overlaps the *ibs* coding sequence and TRD2 overlaps the translation initiation site of *ibs*; interactions with the toxin mRNA occurred through both domains and extended into the surrounding sequences. This likely prevents ribosome binding and translation. *In vivo* analysis suggests that this duplex is likely subject to processing by RNase III; deletion of the *rnc* gene led to increased expression of the toxin *ibs* mRNA ([41], and Fozo, unpublished), implying that degradation is triggered upon duplex formation. Along with this, there are two forms of the Sib antitoxin and deletion of the *rnc* gene impacts accumulation of each form differently. Both forms are complementary to the entire *ibs* coding region and the ribosome binding site; however, the region of complementarity of only the longer form of the Sib sRNA extends into the *ibs* 5' UTR. Deletion of the *rnc* gene leads to accumulation of the longer Sib sRNA and decreased levels of the shorter Sib sRNA [Fozo unpublished]. This indicates that the shorter Sib form may be the result of processing following duplex formation with the *ibs* mRNA. Further work is needed to validate these observations.

Repression of the production of the SymE toxin by the SymR antitoxin also likely includes a translational block [55]. The SymE/SymR toxin-antitoxin pair is not a typical type I toxin-antitoxin locus. Here, the toxic protein SymE is actually quite large at 113 amino acids in length and it is not particularly hydrophobic. It functions similarly to endonucleases, though it has some unusual sequence homology (for more thorough details see [32,55]). SymE overproduction leads to increased mRNA degradation, but the SymR antitoxin can block this toxicity [55].

The SymR RNA is encoded opposite of the *symE* gene, such that the antitoxin RNA would overlap the start codon and ribosome binding site of the *symE* mRNA, suggestive that SymR blocks translation [55]. Mutation of the *symR* promoter increased the levels of the SymE protein by seven-fold, and led to a more modest increase in *symE* mRNA levels. This supports the model that SymR affects *symE* translation and not mRNA stability. Further confirmation came from the comparison of SymE protein levels in a wild type strain and a strain deleted for the *rnc* gene: there were no significant differences in protein levels between the two strains [55]. This data again supports that SymR acts to block translation as opposed to stimulating degradation of *symE*.

A new study recently proposed that the *ralR-ralA* locus of *E. coli* is another type I toxin-antitoxin system [56]. This locus is unusual in that the reported region of complementarity between the toxin and antitoxin lies within the coding region of the toxin mRNA and that the regulation is dependent on Hfq; such features are not found within the previously characterized loci. Initial analysis implies that levels of RalR protein, but not mRNA, are sensitive to the presence of RalA [56]. Although the genetic organization suggests that perhaps the RNAs could interact through overlapping 3' ends, end mapping has not yet been reported. Further work will be needed examining this locus and regulatory control of RalR.

### 2.2. Repressing Type I Toxins: Controlling Degradation and Translation

To date, most type I antitoxins act primarily through a block in translation or increased degradation of the toxin mRNA. There has been limited evidence suggesting that both mechanisms are necessary for an antitoxin to function. However, recent work with the SR4 antitoxin of *B. subtilis* has shown that this RNA is capable of using both approaches [57,58].

The *bsrG*/*SR4* locus is found within the SPβ phage element. Like all type I toxin-antitoxins described to date in Gram-positive bacteria, the toxin mRNA and antitoxin overlap at their 3' ends, and have approximately 120 nt overlap [58]. Analysis by two groups showed that deletion of *rnc* led to elevated levels of *bsrG*, yet it did not seem to be required for repression of the toxin [14,58]. This was suggestive that perhaps the major repressive mechanism by SR4 was not through increasing mRNA degradation.

**Figure 5:**
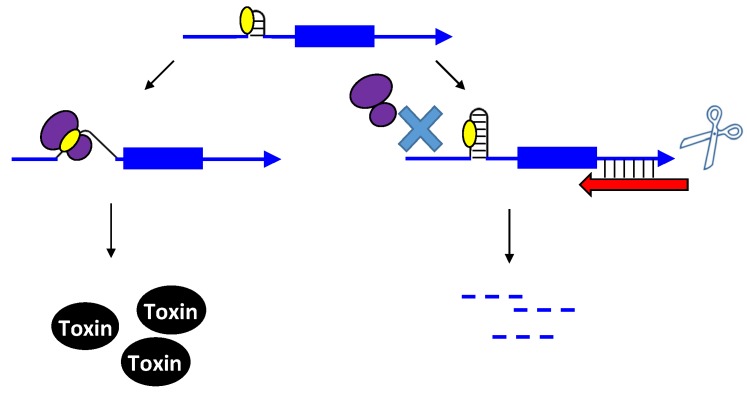
Repression of toxin BsrG production by SR4. The ribosome binding site of the toxin is normally in a 4 nt stem (yellow). Binding by the antitoxin (red) causes structural changes that sequester the ribosome binding site in a longer stem. This interaction with the antitoxin can also trigger toxin mRNA degradation.

To better decipher how SR4 controlled expression of *bsrG*, careful structural analysis was performed both on the toxin and the antitoxin individually as well as the two complexed together. Unexpectedly, upon pairing with SR4, the *bsrG* mRNA underwent significant structural changes quite far from the overlapping 3' ends of the RNAs [57]. Most importantly, there was increased sequestration of the ribosome binding site; analysis of the structure of *bsrG* alone showed that the RBS was in a 4 nt stem, and upon pairing with SR4, the stem was increased to 8 nt (Figure 5). Studies to determine how this altered structure would impact translation proved difficult, but the authors designed a clever assay. They modified the *bsrG* sequence such that it would produce either an 8 nt stem sequestering the ribosome binding site or an 4 nt stem. Only the 8 nt stem construct could be moved into *B. subtilis*; the 4 nt construct was never successfully transformed into the organism [57]. This provided evidence that although SR4 binds far from the translational start of *bsrG*, it can still block translation.

#### 2.2.1. Controlling Protein Expression: mRNA Degradation *versus* Translation Inhibition

While the type I loci examined to date imply that regulation occurs either through toxin mRNA degradation or inhibition of its translation, antitoxins may employ both mechanisms. Regulation by SR4 was carefully examined through a combination of *in vivo* and *in vitro* experiments, which was how the authors could show that a translational inhibition was possible, along with increased mRNA degradation by RNase III [14,57,58]. It may be that other type I antitoxins also can do both, but sufficient data is not yet available.

### 2.3. Type III Antitoxins: Repression through Protein Sequestration

**Figure 6:**
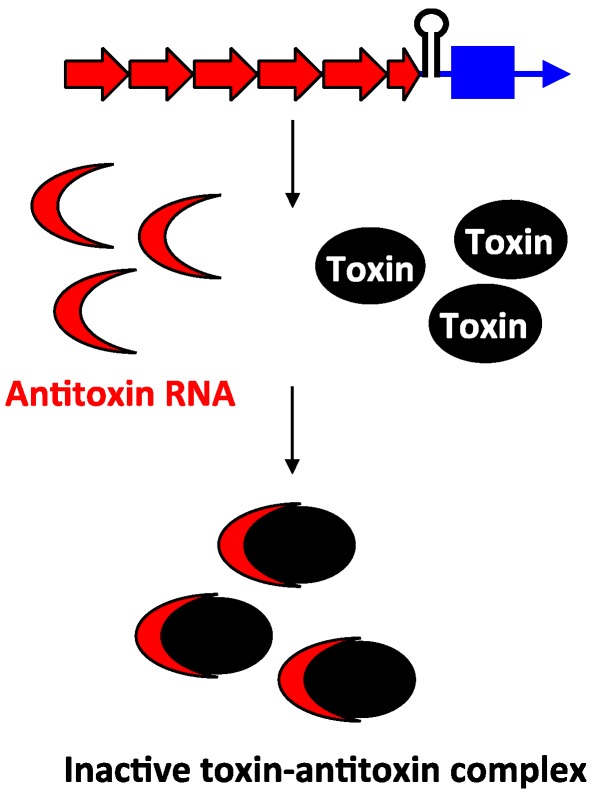
Inhibition of type III toxin activity by antitoxin binding. As exemplified by ToxIN, the antitoxin *toxI* (red) and the toxin *toxN* (mRNA, blue; protein, black) are in a bicistronic operon with a Rho-independent terminator that separates them. The antitoxin (red) RNA ToxI is processed by ToxN, and upon processing, it can bind to preformed ToxN protein.

Unlike the antitoxins of type I loci, the type III antitoxins bind directly to their toxin protein and inhibit the toxin protein’s activity. The first type III toxin-antitoxin system identified was the ToxIN pair in a cryptic plasmid from the Gram-negative bacterium *Pectobacterium atrosepticum* subspecies *atroseptica* [59] (note the species was referred to in the original publication as *Erwinia carotovora* subspecies *atroseptica*). The bicistronic *toxIN* locus contains two genes: *toxI* and *toxN*. The *toxI* gene is composed of 5.5 direct repeats of 36 nt, followed by the *toxN* gene that encodes a 171 amino acid protein, with a Rho-independent transcriptional terminator located in between (Figure 6). The genes are cotranscribed; only a small percentage of transcripts read through the terminator that separates *toxI* and *toxN*. Consequently, *toxI* transcripts are much more abundant compared to *toxN*. When overexpressed in *E. coli*, the ToxN protein caused cell growth stasis. However, this toxicity could be neutralized by co-expression of *toxI*, indicating that the ToxI RNA acts as an antitoxin. Despite the full-length ToxI RNA consisting of 5.5 repeats and being almost 200 nt in length, researchers found that 1–1.5 repeats was sufficient to repress ToxN toxicity [59,60]. Analysis of ToxN revealed that it has ribonuclease activity and processes RNAs including the cognate ToxI antitoxin RNA. Processing of the full-length ToxI repetitive precursor can generate four monomers and crystallographic analysis confirmed that the ToxI monomers physically interact with the ToxN protein. Specifically, a ToxI RNA monomer folds into a pseudoknot structure. Three ToxI monomers bind three ToxN proteins through extensive RNA-protein interactions. This forms a trimeric ToxI-ToxN complex, which leads to inhibition of the ToxN function [60].

Through the use of protein homology searches, as well as the features of type III loci, other homologs to ToxIN and new type III families were identified on plasmids and chromosomes of many bacterial phyla including Firmicutes, Fusobacteria and Proteobacteria [61]. For all cases, the gene pair possesses a putative antitoxin RNA gene consisting of tandem short repeats, followed by a terminator hairpin, and lastly a putative toxin-encoding gene. Moreover, the toxins are predicted to be RNases that cleave their cognate antitoxin RNA precursors. Through these analyses, it was shown that one single bacterium can harbor multiple type III families or multiple copies of the same type III locus [61,62]. These loci, including the AbiQ, the *cptIN*, and *tenpIN* families all share the same genetic organization as the ToxIN module. Thus, these loci are wide spread throughout bacteria.

### 2.4. Function of Type I and Type III Gene Pairs

To date, numerous examples of type I and type III loci have been described in bacterial chromosomes. However, whether these chromosomal copies have a true biological function is still questioned. Given that the first loci were described on plasmids and serve to function in plasmid stabilization, many assumed that these loci are just to maintain “selfish” DNA elements. However, bioinformatic analyses indicate that many chromosomally encoded toxin-antitoxin pairs have no clear homology to mobile genetic elements nor is there evidence for horizontal gene transfer [5]. Also, many toxin gene sequences are highly conserved with strong predicted ribosome binding sites and little evidence of sequence degeneration, suggesting that bacteria are maintaining these genes. This would support the argument that, at least for some chromosomally encoded loci, they do indeed possess a true biological function.

#### 2.4.1. Plasmid Remnants?

The *hok*/*sok* locus of plasmid R1 was the first type I toxin-antitoxin gene pair to be described. Upon cell division, if a daughter cell does not inherit a copy of the plasmid, the unstable Sok antitoxin will be degraded, allowing the stable *hok* mRNA to be translated, and kill the plasmid-less cell. Specifically, the Hok toxin oligomerizes in the inner membrane, causing pore formation, and loss of viability [63]. This is particularly evident with the appearance of “ghost” cells upon Hok overproduction. Hok though appears to act internally as application of exogenous Hok to cells could not induce cell killing; in fact, synthetic Hok could only kill cells if electroporated into the cells [64]. Combined, these data supported that the function of the plasmid encoded *hok*/*sok* locus is to maintain the plasmid within the bacterial population.

With the advent of genomic sequencing, *hok*/*sok* loci were found within the genomes of numerous *E. coli* strains and other Gram-negative bacteria; for some *E. coli* strains examined, there are as many as 12 copies of the locus [5,6]. In some instances, there is evidence of sequence insertions or sequence degeneration, and it is likely that these loci no longer produce the toxin or antitoxin. However, a large number appear to be intact and are potentially expressed [6]. So, what are these chromosomal copies doing? Are they simple remnants of past plasmids? Do they serve to maintain plasmids within the population or maintain chromosomal stability in the population? Given that there are numerous copies within a single species and some loci appear degenerate, these questions are not trivial to address. Future work will hopefully resolve whether or not chromosomally encoded *hok*/*sok* gene pairs have a true biological function.

#### 2.4.2. Impairment of Chromosomal Structure

As described above, the RNA I/RNA II locus of the pAD1 plasmid of *E. faecalis* confers plasmid addiction through post-segregational killing [65]. Like Hok and many other type I toxins, the Fst toxin, encoded by RNA I, also contains a putative transmembrane domain. When overexpressed, it can cause cell membrane permeabilization and cessation of macromolecular synthesis [46]. However, unlike Hok, overproduction of Fst does not result in the formation of ghost cells (reviewed in [66]). A recent study revealed that the Fst-resistant *E. faecalis* M7 strain harbors a mutation in the *rpoC* gene encoding the β subunit of RNA polymerase [67]. Normally, an Fst-sensitive *E. faecalis* strain will induce the expression of a variety of membrane transporters upon exposure to Fst; however, the M7 strain did not. This suggested that Fst induces membrane transporters and that induction may deplete the cell of energy. To validate this, the authors treated wild type *E. faecalis* with reserpine, an inhibitor of transporters, and then exposed the treated strain to Fst. Upon treatment with reserpine, wild *E. faecalis* survived Fst exposure far better than without [67]. Thus, the induction of membrane transporters is a major cause of Fst-induced toxicity.

Intriguingly, Fst induced membrane damage occurs late upon Fst overproduction [68]. This indicated that the loss of membrane integrity may not be the primary effect of Fst toxin. Studies have revealed that the primary effect of Fst overexpression is condensation of the nucleoid. The effects on nucleoid structure have been observed not only in *E. faecalis*, but also in *Staphylococcus aureus*, *B. subtilis*, and *E. coli*; although how Fst disrupts nucleoid structure remains unclear [7,46,68]. Interestingly, bioinformatic analysis concluded that the Fst and Ldr proteins are within the same toxin superfamily [5]. Overexpression of LdrD led to a similar nucleoid condensation in *E. coli*, suggesting that this is a conserved feature for this family [34].

Like the *hok*/*sok* locus, numerous homologs to RNA I/RNA II have been identified in many bacterial chromosomes [5,7]. Given the above effects on membrane transportation upon Fst overproduction, it is interesting to note that several chromosomal RNA I/RNA II loci are flanked by transporters, specifically, carbohydrate transporters [7]. Also, for those examined, both the toxin and antitoxin are transcribed, indicative that they are likely functional. What their biological role may be is not clear. For a homolog identified in *Streptococcus mutans*, described below, there are links to persister cell formation [51]. For a homolog found on the chromosome of *E. faecalis* V583, deletion of the antitoxin component (RNA II) led to increased virulence in a mouse model, though how RNA I could contribute to virulence is not known [69]. It is important to note that even very low levels of Fst production can lead to the formation of suppressors, so further examination of this RNA II deletion strain is needed. Interestingly, a homolog of RNA I/RNA II in *S. aureus* indicates that expression of the toxic peptide is induced upon cellular stress, suggesting that it may have a role in responding to changing environments [53].

#### 2.4.3. Persister Formation

Although a bacterial population maybe genetically identical, cellular growth and gene expression are not equivalent across the population of cells. A small portion of cells within a population are non-growing or slow-growers; these “dormant” cells are termed persisters [70,71]. Given that most antibiotics target actively growing cells, persisters are often highly antibiotic resistant [72]. The antibiotic ciprofloxacin targets bacterial DNA gyrase, leading to DNA damage and induction of the SOS response by relieving repression of genes controlled by the transcription factor LexA [73]. The type I toxin gene *tisB* is induced by SOS damage [39]. Deletion of the entire *tisB*/*istR-1* locus in *E. coli* led to a decrease in persisters tolerant to ciprofloxacin. Yet a strain deleted for only the antitoxin IstR-1 exhibited a 10- to 100-fold increase in the level of persisters [74,75]. This indicated that TisB, even expressed at its endogenous level, plays an essential role in persister formation.

How then could a small hydrophobic protein led to persister formation? A previous study demonstrated that an epitope tagged version of TisB localized to the inner membrane of *E. coli* [76]. Work with synthetic TisB and vesicles showed that TisB monomers can rapidly and spontaneously bind to membranes [77]. It is thought that these monomers within the membrane may form dimers that would allow protons to pass. Analysis by another group suggested that TisB monomers aggregate to form pores that are selective for anions [78]. Regardless, production of TisB and its insertion in the membrane would cause a loss of proton motive force and a decline of intracellular ATP; indeed overproduction of TisB for only five minutes led to a dramatic loss of ATP within *E. coli* [76]. Ciprofloxacin requires ATP, thus expression of TisB would cause a decrease in ATP levels leading to both an inhibition of macromolecule synthesis and an inhibition of the antibiotic’s activity. So by inserting into the inner membrane and causing a loss in proton motive force, leading to subsequent depletion of ATP for ciprofloxacin activity and macromolecule synthesis, TisB production can increase the number of persisters within the population [74,75,79].

This consequential decrease in cellular metabolism caused by TisB is similar to what has been reported for type II toxins. Type II toxin proteins are often “larger” (~100 amino acids) than type I toxins and are not particularly hydrophobic. They have defined biochemical activities with most examples functioning as either ribonucleases or inhibitors of DNA gyrase [1]. Deletion of multiple type II toxin-antitoxin loci in *E. coli* decreased persister formation [80]. It was hypothesized that by induction of type II toxin activity (specifically ribonuclease activity), cellular translation would be halted, giving rise to the “dormant” persister cell phenotype. A recent study utilizing chemical inhibitors of macromolecular synthesis supports this: by decreasing macromolecular synthesis chemically, the frequency of persister cell formation was far higher over control populations that were not treated [81]. Thus, type II toxins and TisB appear to induce persister formation by reducing cellular metabolism through the inhibition of macromolecular synthesis. It is critical to note, however, that most identified type I toxins have not been examined to conclude whether or not they participate in persister formation, but such analysis would be of great interest.

The studies with TisB illustrate another important point: the critical balance of toxin levels within a cell. Overproduction of TisB clearly causes cell death [39,41,76], yet in some instances, it can lead to persister formation [74]. In particular, analysis suggests that the expression of *tisB* is induced 1000-fold under SOS conditions yet this does not induce cell killing [74,76]. Thus, the fine-tuning of toxin and antitoxin levels is critical for persister formation. When the toxin level is below that of the antitoxin, the antitoxin successfully represses toxin expression, and the cell is unharmed. If the toxin level is higher than that of the antitoxin, the toxin escapes repression and impairs cell growth. Depending upon how excessive toxin levels are to the levels of the antitoxin will determine whether the cell becomes dormant (*i.e.*, persister) or is killed. When the levels of the toxin and the antitoxin are close, some cells will have an imbalance in the ratio of toxin: antitoxin. Owing to the stochastic nature of bacterial populations, this small portion of the population will become persisters [82]. The exact amounts of TisB needed to form ciprofloxacin-induced persisters is currently not known but will be of great interest in understanding the balance between the benefits and costs of possessing this toxic gene.

In the Gram-positive bacterium *S. mutans*, a homolog to the RNA I/RNA II locus of pAD1 has been linked to persister cell formation [51]. Deletion of this locus, known as Fst-Sm/srSm, did not impact persister formation. However, the number of persisters was greatly reduced upon treatment with cell-wall damaging antibiotics in a strain harboring the entire locus on a multicopy plasmid [51]. Unlike *tisB*, this observation appears to be due to increasing the number of Fst-Sm/srSm copies within the cell; analysis has not revealed whether or not this observation can be attributed either to the toxin, the antitoxin, or just increased copies of the entire locus. How then, would increasing the number of copies of this locus lead to decreased persister cell formation? This could be due again to levels of the toxin *versus* the antitoxin; by increasing the number of loci within the cell, there could be an imbalance in the ratio of toxin: antitoxin, leading to heighten cell killing and decrease in persister formation. A detailed analysis of toxin and antitoxin RNA and toxin protein levels across the cellular population will be needed to further conclude how this gene pair impacts persister cell formation.

#### 2.4.4. Chromosomal Stabilization and Recombination

In *B. subtilis*, many of its predicted chromosomally encoded type I loci are located within integrated mobile elements. For instance, the *txpA*/*ratA* and *bsrH*/*as-bsrH* loci are located within the *skin* element [15,83,84], while *bsrG*/*sr4* and *yonT*/*as-yonT* are within the SPβ prophage [5,58]. Overexpression of these toxin genes slows cell growth or causes cell lysis, while deletion of these genes does not lead to noticeable consequences.

The presence of these gene pairs on mobile genetic elements suggests that they may maintain these elements within the chromosome. This maintenance may then provide the cell with a selective advantage under specific environmental conditions. For example, studies of the SPβ prophage suggest that it contains genes that are beneficial to *B. subtilis*. The gene *sspC* encodes a small acid-soluble protein that provides high UV light resistance to spores and is found on the SPβ prophage [85]. The gene *nonA*, examined in the SPβ prophage region of the *B. subtilis* Marburg strain, encodes a protein that protects cells against infection from the bacteriophage SP10 [86]. Perhaps the two toxin-antitoxin loci then contribute to maintaining this element within the population. Interestingly, the BsrG type I toxic protein that is encoded on the SPβ prophage is temperature-sensitive. Rapid degradation of *bsrG* mRNA was observed at 48 °C, indicating a potential role of this toxin in response to changing temperatures [58].

However, some prophage regions may simply act as selfish elements in the chromosome. One possible example for this theory is the *skin* element of *B. subtilis*. This large element (48 kb) is located within the *sigK* gene encoding the RNA polymerase sigma factor σ^K^ [87]. During sporulation, *skin* is excised from the chromosome, creating an intact *sigK* gene. This excision only occurs from the chromosome of the mother cell, and not from the chromosome of the forespore. An engineered strain with the *skin* element deleted was able to grow and sporulate normally, suggesting that this region does not play an essential role in viability or sporulation [87]. Thus, is *skin* simply a piece of selfish DNA? Within the *skin* are two different type I toxin-antitoxin loci: *txpA*/*ratA* and *bsrH*/*as-bsrH*. Thus, if the *skin* is lost, expression of those toxins could be lethal for the cell, helping to maintain the element within the population. However, a study did identify genes conferring arsenate and arsenite resistance within the *skin* element [88], implying that there are genes of value to *B. subtilis* within this region. Perhaps a more detailed analysis of the genes encoded with the *skin* element could determine if the function for *txpA*/*ratA* and *bsrH*/*as-bsrH* is to maintain a “useful” piece of mobile DNA.

It is also possible that these type I modules of *B. subtilis* have functions other than stabilizing the chromosomal regions. It has been shown that several type I toxin encoding genes, such as *brsG*, *brsE* and *brsH*, have putative ResD response regulator binding sites upstream, indicating that their toxin products may participate in response to oxygen limitation [89]. Durand et al noted that the described toxins and antitoxins are all under control of the vegetative sigma factor σ^A^; thus all could be quickly induced [89]. Further analysis of the balance between the expression of the toxins and antitoxins will provide much-needed evidence for their physiological roles.

In *E. coli*, the newly described DinQ/AgrB locus may also play a role in chromosomal stability [42]. Transcription of the *dinQ toxin*, like *tisB* and *symE*, is also regulated by LexA, and is induced upon DNA damage. Deletion of the antitoxin *agrB* led to elevated DinQ levels; however, this deletion did not directly impact SOS activities. Instead, a strain deleted for *agrB*, had a 400-fold reduction in recombination frequency, suggesting that DinQ interferes with recombination [42]. The small toxin is found in the membrane, and its overproduction can lead to decreased ATP levels, like TisB, as well as increased nucleoid condensation, like Fst and LdrD. Further work to elucidate how these phenotypes are linked to DinQ biochemical activity will be of great interest.

#### 2.4.5. Protection from Foreign DNA

In 1996, researchers showed that when the *hok*/*sok* locus was cloned onto a high copy plasmid, *E. coli* was better protected from infection by the phage T4, suggesting that toxin-antitoxin loci can protect bacterial populations from foreign DNA [90]. In fact, all of the described type III toxin-antitoxin loci serve as phage abortive infection (*abi*) systems and confer phage resistance at the population level. Essentially, the type III toxin is active during phage infection and causes death of the infected cell prior to the release of phage progeny [91]. The toxin possesses ribonuclease activity and is thought to cleave RNA (host and phage) leading to cellular damage and death. Interestingly, it appears that this mechanism is a rather “generic” means of protection from numerous different phages. For example, the ToxIN locus protected *P. atrosepticum* from 13 examined phages, and when moved into *E. coli*, it was able to protect the non-native host from 5 coliphages [59]. The AbiQ system also conferred resistance to at least 3 lactcoccal phage groups [92].

How type III toxin activity is triggered by phage infection is not clear. For ToxIN of *P. atrosepticum* and AbiQ of *Lactococcus lactis*, it appears that both the toxin and antitoxin are constitutively transcribed through cell growth [59,62]. Yet prior to phage infection, the toxin must be sequestered by the antitoxin to prevent unnecessary cell death. Upon phage infection, a sufficient amount of functional toxic protein needs to accumulate to trigger cell death. For ToxIN, it has been suggested that during the course of phage infection, host transcription or translation maybe disrupted, resulting in an imbalance of ToxI and ToxN. These imbalances lead to the accumulation of free ToxN, which is then able to degrade RNA, and eventually, lead to cell death [59].

For the other well-characterized type III loci, this is likely not the case. The AbiQ locus is found on the native plasmid pSRQ900 of *L. lactis*. Here, the toxin gene is referred to as *abiQ* (ABIQ for the protein) and antitoxin gene is *antiQ*. Analysis of this locus found that *abiQ* mRNA levels decrease over time during phage infection, which was attributed to cell death triggered by the activity of the ABIQ toxin [62]. Yet, no significant change in the abundance of ABIQ toxin was observed, suggesting that phage does not play an important role in increasing the amount of ABIQ toxin. Moreover, levels of the antiQ RNA remain constant, though whether the RNA is still bound to the ABIQ toxin is not clear. These data indicate that the cell-killing effect caused by this type III toxin is likely due to a functional switch from “silent” to “active”, rather than a change in levels upon infection. Perhaps then the phage may activate the ABIQ protein or sequester the antiQ RNA, leading to accumulation of free functional ABIQ inside the cell [62]. Further studies regarding the kinetics of the toxin-antitoxin interaction and analysis of their activities are needed to understand how activation of the type III toxin occurs during phage infection.

#### 2.4.6. Inhibition of Competitors

The *txpA*/*ratA* homologous locus, *sprG1*/*sprF1*, in *S. aureus* was recently described [93]. Like the original *txpA*/*ratA* locus, the locus characterized was found on a mobile genetic element, ΦSa3 PI, a phage integrated within the chromosome; however, additional copies were predicted within the core genome [14,15,93]. The *sprG1* gene encodes two toxic peptides from two in-frame initiation codons, and the *sprF1* gene encodes an antitoxin sRNA. Interestingly, we note that even the original member of this family, *txpA* of *B. subtilis*, may produce two peptides (see [15]). For *S. aureus*, overproduction of either the long or short SprG1 peptide inhibited cell growth and caused cell death; however, co-expression of the SprF1 antitoxin could repress toxicity induced by either toxic peptide [93]. Both SprG1 peptides contain a putative transmembrane domain and both proteins were detected in the supernatant. Given this, the authors investigated whether or not external addition of either peptide could be toxic to cells. Application of synthetic forms of SprG1 inhibited the growth of *E. coli*, *Pseudomonas aeruginosa* and *S. aureus*, as well as induced lysis of eukaryotic cells [93]. Interestingly, the longer synthetic peptide was more effective against human erythrocytes and the shorter variant more active against bacterial isolates. While this is the first type I toxin shown to be able to induce toxicity when applied externally to cells, more analysis will be needed to confirm that sufficient SprG1 is produced and released from cells to cause killing under native, endogenous conditions.

#### 2.4.7. Nucleic Acid Cleavage

Almost all type I toxins described to date have a putative transmembrane domain that is thought to contribute to membrane damage upon their production. However, both SymE and RalR do not follow this paradigm. Overproduction of both SymE (SymE/SymR) and RalR (RalR/RalA) toxins were toxic to *E. coli*, yet co-expression of their cognate antitoxin (SymR or RalA) could prevent this toxicity [55,56]. SymE, is a rather “large” protein for a type I toxin at 113 amino acids in length. SymE acts as a ribonuclease and its overproduction triggers mRNA degradation (for a more thorough discussion of the unusual homology of SymE see [32,55]). Interestingly, SymE, like TisB and DinQ, is also induced by DNA damage and under the control of the LexA promoter. Thus, three type I toxins of *E. coli* are triggered by the SOS response, signifying the importance of DNA stability and repair for cell survival.

The RalR toxin is encoded on the *rac* prophage in the genome of *E. coli* along with its cognate antitoxin-encoding gene *ralA* [56]. RalR was shown to cleave DNA in an *in vitro* assay, but not RNA, suggesting it functions as a DNase. Deletion of either *ralR* alone or *ralR*/*ralA* locus in *E. coli* resulted in greater sensitivity towards fosfomycin, an antibiotic that inhibits peptidoglycan biosynthesis [94,95]. It will be interesting to see how the DNase activity of RalR could lead to increased resistance to a cell wall synthesis inhibitor.

## 3. Regulating the Toxin mRNA *versus* the Protein

Although type I and type III toxins are repressed by RNA antitoxins, the mechanisms of repression are distinctly different. In the type I system, the antitoxin sRNA targets the mRNA of the toxin and impairs the translation and/or the stability of the toxin mRNA, thus decreasing production of the toxic protein. In the type III system, the antitoxin RNA directly binds to the toxin protein to inhibit its activity. The differences regarding the modes of action of the antitoxin RNAs raise the question—why does toxin repression occur at different levels?

### 3.1. Type I Antitoxins: Base Pairing to the Toxin mRNA

Most type I toxins possess a transmembrane domain, suggesting that these small proteins can insert into the cell membrane. Indeed, it has been shown that the *E. coli* TisB toxin can rapidly and spontaneously bind to the membrane once translated, and the TxpA homolog of *S. aureus* (SprG1) is found within the membrane and cellular supernatant [76,77,93]. Therefore, if the repression of the toxin requires binding of the antitoxin, it may be difficult for the antitoxin to “catch” the small proteins before they “hide” inside the membrane. However, by targeting the toxin mRNA, the type I antitoxin can completely turn off toxin production, which may be more efficient in blocking possible pore formation. Also, the general function of type I toxin-antitoxin gene pairs may be adaptation to different stress conditions. For example, the TisB/IstR, SymE/SymR and DinQ/AgrB pairs in *E. coli* are SOS-induced, while the RNA I/RNA II homolog of *S. aureus* (SprA1/SprA1_AS_) is induced in response to acidic and oxidative stress [39,42,53,55]. Thus, under normal, favorable growth conditions, toxic protein production is unnecessary. Consequently, regulating the toxins at the mRNA level via the antitoxin RNA could not only save energy from producing a protein that is not needed, but would also limit any possible damage from unwarranted toxin translation.

### 3.2. Type III Antitoxins: Binding to the Toxic Proteins

While the function of the chromosomal type I loci remains unresolved (and note that there may be a separate function for each unique family), the type III gene pairs described to date act as abortive phage infection (*abi*) systems. Upon phage infection, the type III toxin promotes death of the infected bacterial cell, preventing release of mature phage, thereby limiting phage infection within the population. Due to the incredible abundance and population dynamics of phages, bacteria constantly face threats of phage predation [96]. Furthermore, some have speculated that each individual bacterial species could be susceptible to at least 10 different phage species [97]. Additionally, phage replication is much higher than that of bacteria. A filamentous phage can undergo 6 replication cycles per min, while lambda can double its DNA every 2–3 min [98,99]. In order to maintain the bacterial population, bacterial cells must rapidly respond to phage infection.

Consequently, by having a pool of pre-made type III toxin proteins within the cell, a bacterial cell is poised to quickly handle phage infection. Regulation of type III toxins after translation likely ensures rapid activation of the *abi* response as the toxins could be rapidly released from sequestration and cause cell death. As was described above, upon formation of the RNA duplex between a type I toxin-antitoxin pair, the toxin mRNA will often be degraded. Thus, repression by type I antitoxins is usually irreversible. However, repression by the type III antitoxins is reversible, and does not impact the levels of the toxic protein. This again can contribute to the quick response to phage infection. One may argue that this reversible regulation is risky, since any “leaky” type III toxins may cause detrimental effects to the cell. However, given the bicistronic nature of type III loci, antitoxin RNA levels were shown to be remarkably greater than that of the toxin. For example, approximately 90% of the transcripts of the *toxIN* locus terminate at the Rho-independent terminator [59]. The ToxI: ToxN ratio would then be about 10:1, which would allow for sufficient repression of the ToxN toxin. Additionally, it has been shown that the antiQ RNA antitoxin remains at a similar level throughout phage infection, suggesting this RNA is very stable or constitutively produced [62]. These observations suggest that type III toxin repression is tight and that the irreversible nature of its repression likely does not pose a problem for the cell.

## 4. Conclusions

There are numerous toxin-antitoxin loci found within bacterial chromosomes; the type I and type III pairs represent only a fraction of what has been described to date [1]. The type I and type III loci are unique in that they utilize RNA as antitoxins, yet they use their RNA antitoxins very differently, illustrating the immense versatility of RNA as a regulatory molecule. It is important to note that to date these antitoxins have been shown to act either through interaction with toxin mRNA or protein. It remains to be seen if an antitoxin RNA can act by interacting with both the toxin mRNA and toxin protein. For example, the small RNA McaS of *E. coli* was shown to act via base pairing to target mRNAs and act by binding to the protein CsrA [100,101]. It is intriguing to think an antitoxin RNA may be identified that can act on both RNA and protein. It has been also been demonstrated that some of the type II antitoxins not only repress their cognate toxins, but also control the expression of several other genes [102,103,104,105,106]. As many type I antitoxins are found at much higher levels than their toxin counterparts, could some of the type I antitoxins have additional cellular functions? Finally, the role of so many type I toxin-antitoxin loci remains elusive; given their broad distribution and conservation throughout a variety of species, much more work deciphering the biochemical activity of the toxins, the physiology of deletion strains, and thorough analysis of gene expression control will provide the answer to the question “what are all of these loci doing?”
